# The Effects of *Deyeuxia purpurea* Wetland Degradation on Plant Communities and Key Soil Factors in the Sanjiang Plain

**DOI:** 10.3390/plants15060918

**Published:** 2026-03-16

**Authors:** Chuncheng Ou, Haipeng Dong, Xin Sui, Tingting Fu, Yingnan Liu, Haixiu Zhong, Yu Zhang, Jiawen Liang, Xuwen Hou, Hongwei Ni, Lihong Xie, Jifeng Wang

**Affiliations:** 1 Institute of Natural Resources and Ecology, Heilongjiang Academy of Sciences, Harbin 150040, China; ouchuncheng0105@163.com (C.O.); dhp971112@163.com (H.D.); liuyingn234@163.com (Y.L.); zhx971030@163.com (H.Z.); lengning1029@126.com (Y.Z.); 18845728229@163.com (J.L.); houxuwen0209@163.com (X.H.); 2 Heilongjiang Provincial Key Laboratory of Ecological Restoration and Resource Utilization for Cold Region, School of Life Sciences, Heilongjiang University, Harbin 150080, China; xinsui_cool@126.com; 3 College of Modern Agriculture and Ecological Environment, Heilongjiang University, Harbin 150080, China; futingting1128@163.com; 4 Heilongjiang Academy of Forestry, Harbin 150040, China; nihongwei2000@163.com

**Keywords:** *Deyeuxia purpurea* wetland, wetland degradation, plant community characteristics, species diversity, key soil factors

## Abstract

The succession of plant communities and soil-driven mechanisms triggered by wetland degradation are central issues in global ecology. To investigate the effects of *Deyeuxia purpurea* wetland degradation on plant community characteristics and its key soil regulatory factors, this study selected *D. purpurea* wetlands with different degradation degrees in the Sanjiang Plain as research objects and analyzed the characteristics of plant communities, soils, and their relationships. The results indicated that wetland degradation was significantly associated with turnover in plant community composition, with hydrophytic species progressively replaced by mesophytic and xerophytic species. As degradation intensified, Simpson’s diversity index, the Shannon–Wiener index, Pielou’s evenness index, and Patrick’s richness index all increased significantly. The non-degraded wetland exhibited significantly higher aboveground, belowground, and total biomass than the degraded wetlands. Aboveground and total biomass showed a significant negative correlation with the diversity index. Soil pH, water content (WC), total phosphorus (TP), dissolved organic nitrogen (DON), and ammonium nitrogen (NH_4_^+^-N) were key factors associated with changes in plant community diversity and biomass. Partial least squares path modeling (PLS-PM) and variance partitioning analysis (VPA) further quantified potential association pathways, showing that wetland degradation exerted both direct and indirect effects on key soil physicochemical factors and plant community characteristics. Specifically, wetland degradation was directly associated with decreases in soil pH, WC, and TP, while positively affecting soil dissolved organic nitrogen (DON) and plant diversity. It also indirectly influenced plant species composition and biomass through changes in soil pH, WC, DON, and TP. TP was negatively correlated with plant diversity and biomass, whereas ammonium nitrogen had a direct positive effect on species composition. Dissolved organic nitrogen directly negatively affected species composition. Overall, this study systematically elucidates plant community response patterns and the synergistic driving mechanisms of multiple soil factors during *D. purpurea* wetland degradation, providing an important scientific basis for wetland conservation and ecological restoration in the Sanjiang Plain.

## 1. Introduction

Wetlands are among the ecosystems with the highest ecological value and service functions worldwide. Although they cover only 6–8% of the Earth’s land surface [1,2], wetlands support more than 40% of global species diversity and contribute approximately 41–43.5% of the total ecosystem service value, amounting to nearly USD 50 trillion annually. They play irreplaceable roles in carbon sequestration, climate regulation, water purification, and biodiversity conservation [3,4,5,6]. However, in recent years, intense human disturbances, including urban expansion, agricultural reclamation, and overexploitation of water resources [7], together with accelerating global climate change and environmental degradation [8,9], have driven widespread wetland loss and degradation worldwide, severely threatening wetland ecosystem structure and function.

Wetland degradation generally refers to the sustained decline in ecosystem structure, function, and services under natural or anthropogenic pressures. It is mainly manifested by altered hydrological conditions, deterioration of soil physicochemical properties, reduced vegetation cover, biodiversity loss, and increased landscape fragmentation [3,9]. As a core component of wetland ecosystems, plant community structure and function serve as key indicators of wetland health, and their successional dynamics directly reflect wetland degradation and recovery processes [10]. Previous studies have shown that wetland degradation often induces a shift in dominant plant species from hydrophytic or aquatic species to mesophytic and xerophytic species, thereby weakening the original ecological functions of wetlands [11]. Even following restoration, plant community diversity and composition often fail to return to natural states in the short term, suggesting that degradation may impose long-lasting impacts on ecosystem functioning [12]. Importantly, the effects of wetland degradation on plant community characteristics are highly ecosystem-specific, with distinct patterns observed among different wetland types (e.g., saline–alkali inland wetlands, alpine lakeshore wetlands, and semi-arid wetlands) [13,14,15]. This highlights the need for degradation mechanism studies tailored to specific wetland types.

Plant community characteristics and dynamics are strongly regulated by soil environmental conditions [16]. Wetland degradation is often accompanied by soil moisture loss, which triggers cascading changes in key soil physicochemical properties, such as soil pH and nutrient availability (e.g., nitrogen and phosphorus). These changes ultimately shape plant community structure, biomass allocation, and diversity patterns by influencing plant resource acquisition strategies, productivity, and interspecific competition [17,18,19]. For example, a marked decline in soil moisture content during wetland degradation is widely recognized as a primary driver of wetland deterioration [20]. At the same time, degradation leads to declining soil quality and reduced concentrations of essential nutrients, including organic carbon, nitrogen, and phosphorus [18,19]. Reduced soil moisture directly affects plant biomass, cover, and height [17], while indirectly constraining community regeneration and recovery potential through its effects on soil pH and seed bank diversity [21,22]. Soil moisture and nutrient availability jointly regulate plant community structure, biomass distribution, and functional diversity by shaping plant productivity and community assembly processes [23,24]. Despite substantial research on changes in plant communities and soil properties during wetland degradation, the coupled relationships between these components—particularly which soil factors act as key drivers of plant community succession and how they exert direct and indirect effects—remain critical scientific questions requiring further investigation.

The Sanjiang Plain is the largest concentrated area of freshwater marsh wetlands in China. Over the past two decades, however, extensive agricultural development has resulted in substantial losses of natural wetlands. Agricultural irrigation has diverted large amounts of water resources originally sustaining wetland ecosystems, intensifying wetland fragmentation and markedly reducing ecological functions, which has led to pronounced changes in wetland plant community characteristics [25,26]. *Deyeuxia purpurea*, a widespread and dominant species in Sanjiang Plain wetlands, is highly sensitive to environmental change and thus represents an ideal indicator species for assessing wetland degradation processes [27]. In this study, *D. purpurea* wetlands along a gradient of degradation in the Sanjiang Plain were investigated to (1) characterize patterns of variation in plant community composition, diversity, and biomass under different degradation levels; (2) identify key soil factors driving changes in plant community diversity and biomass; and (3) quantify the coupling relationships among wetland degradation, key soil factors, and plant community characteristics. The results are expected to enhance understanding of the ecological processes underlying wetland degradation and to provide a theoretical basis for wetland conservation, restoration, and management in the Sanjiang Plain and similar regions.

## 2. Results

### 2.1. Characteristics of Plant Community Composition Under Different Degradation Stages of D. purpurea Wetlands

Along the wetland degradation gradient, the importance value of *D. purpurea* decreased significantly from 660.46 in ND to 182.88 in HD. Indicator species analysis (Table 1) revealed the following significant indicator species: in ND, *Carex miyabei* var. *maopengensis* (IV = 65.02), *Galium trifidum* (IV = 14.52), and *Carex pseudo-conica* (IV = 19.72), all typical wetland plants; in LD, *Lysimachia thyrsiflora* (IV = 23.39); in MD, *Stellaria radians* (IV = 42.63) and *Nelumbo lutea* (IV = 32.39); and in HD, *Geranium platyanthum* (IV = 67.98), *Onoclea sensibilis* var. *interrupta* (IV = 110.56), *Thelypteris palustris* (IV = 59.39), *Deyeuxia neglecta* (IV = 62.94), *Filipendula* × *intermedia* (IV = 41.12), *Galium kamtschaticum* (IV = 16.96), and *Veratrum dahuricum* (IV = 27.58). These results clearly reflect a successional trend from wetland-dominated species to dryland-dominated species (Table 1).

**Figure 1:**
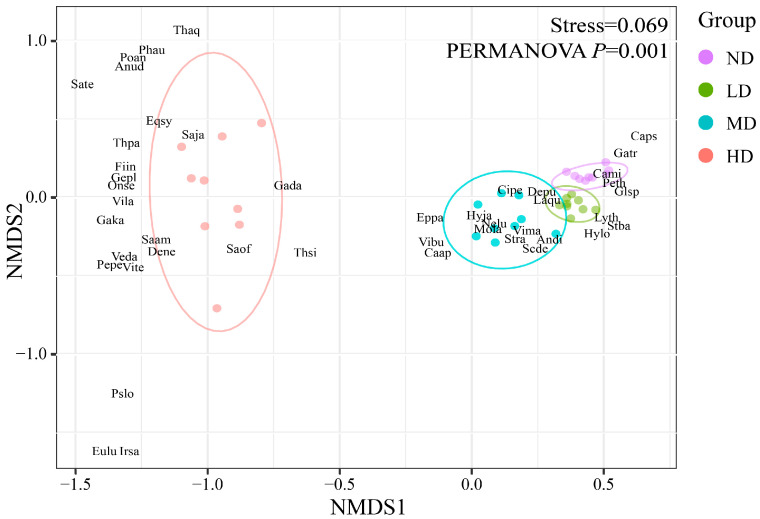
Non-metric multidimensional scaling (NMDS) of *D. purpurea* communities along the degradation gradient in Sanjiang Plain wetlands. ND, non-degraded; LD, slightly degraded; MD, moderately degraded; HD, heavily degraded. Species abbreviations consist of the first two letters of the genus and species names, as detailed in Table 1.

NMDS results revealed changes in species composition of *D. purpurea* communities along the degradation gradient, with a Stress value of 0.069. The first axis (NMDS1) represents the direction of degradation reversal from left to right, with plots arranged from severely degraded to undegraded wetlands, indicating that the degradation gradient is the primary factor driving differentiation in species composition. Regarding plot distribution, undegraded and slightly degraded wetlands showed a concentrated pattern, whereas moderately and heavily degraded wetlands exhibited a more dispersed pattern, indicating greater variation in species composition in the latter. In terms of species distribution, most species in undegraded and slightly degraded wetlands were relatively evenly distributed across plots, while species in moderately and heavily degraded wetlands were confined to specific plots. Heavily degraded wetlands displayed the greatest separation from other groups, representing a key gradient affecting species composition. Overall, these results reflect changes in both common and rare species within communities along the degradation gradient. Permutational Multivariate Analysis of Variance (PERMANOVA) confirmed the statistical significance of the above differences, showing highly significant variations in community composition among different degradation gradient groups (R^2^ = 0.737, *p* = 0.001). This indicates that the degradation gradient explains 73.7% of the total variation in community composition, exerting a strong effect on species composition. Overall, these results reflect the changes in the composition of common and rare species in communities along the degradation gradient (Figure 1).

### 2.2. Characteristics of Plant Community Species Diversity and Biomass Under Different Degradation Stages of D. purpurea Wetlands

**Figure 2:**
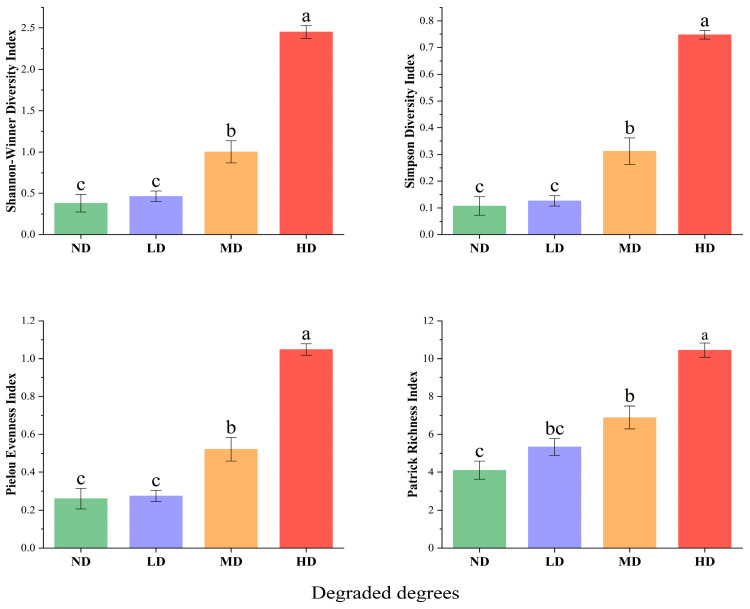
Variation in plant community diversity along degradation gradients. ND, non-degraded; LD, slightly degraded; MD, moderately degraded; HD, heavily degraded. Data are presented as mean ± standard error (SE). Different letters indicate significant differences between treatments (*p* < 0.05).

The plant community diversity indices for degraded wetlands in the study area are shown in Figure 2. Wetland degradation significantly influenced the Shannon–Wiener index, Patrick’s richness index, Pielou’s evenness index, and Simpson’s diversity index (*p* < 0.05). All four indices increased progressively with the intensification of wetland degradation. HD wetlands exhibited the highest diversity, with all indices significantly greater than those in ND, LD, and MD wetlands (*p* < 0.05). In contrast, ND wetlands had the lowest diversity, with all indices significantly lower than those in MD and HD wetlands (*p* < 0.05).

The community similarity index (I) was used to assess differences between wetlands with varying degrees of degradation and the reference system, which in this study was the ND *D. purpurea* wetland (Table 2). The Sørensen similarity index decreased progressively as degradation intensified. In HD wetlands, the community similarity was 14.63%, indicating that only 14.63% of species were shared with ND wetlands. The remaining 85.37% of species had been replaced by other drought-tolerant species during the successional process associated with degradation.

**Figure 3:**
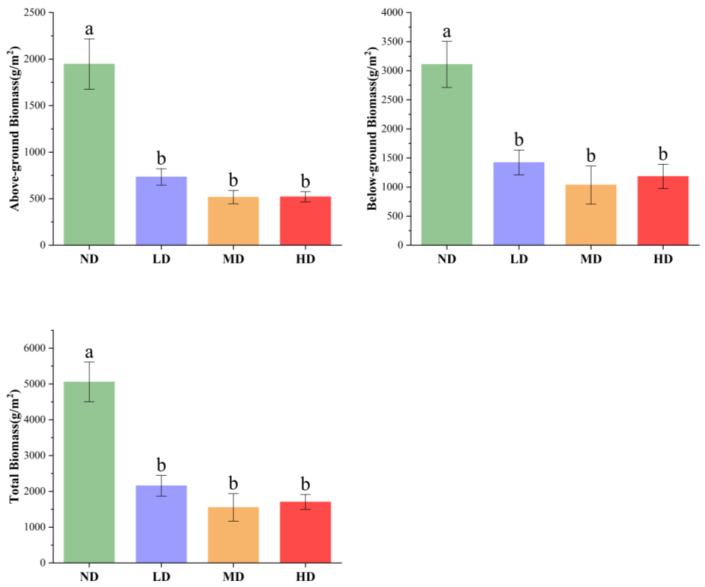
Aboveground biomass (AGB), belowground biomass (BGB), and total biomass (TB, dry weight) of wetland plant communities under different degradation stages. ND, non-degraded; LD, slightly degraded; MD, moderately degraded; HD, heavily degraded. Data are presented as mean ± standard error (SE). Different letters indicate significant differences between treatments (*p* < 0.05).

As shown in Figure 3, the aboveground, belowground, and total biomass of wetland plants gradually declined with increasing degradation. Biomass in ND wetlands was significantly higher than in all degraded wetlands (*p* < 0.05). However, no significant differences in aboveground, belowground, or total biomass were observed among LD, MD, and HD wetlands (*p* > 0.05).

### 2.3. Correlation Analysis Between Plant Community Species Diversity and Biomass in D. purpurea Wetlands

**Figure 4:**
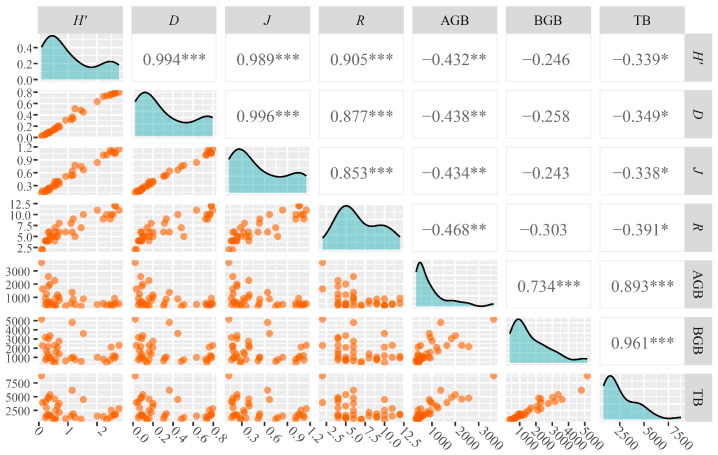
Pearson correlation between biomass and diversity indices of plant communities. Diagonal: density plots showing the distribution of individual variables; lower triangle: scatter plots for row–column variable pairs, illustrating relationships between variables; upper triangle: Pearson correlation coefficients with significance levels (* *p* < 0.05; ** *p* < 0.01; *** *p* < 0.001). *H′*, Shannon–Wiener diversity index; *D*, Simpson’s dominance index; *J*, Pielou’s evenness index; *R*, Patrick’s richness index; AGB, aboveground biomass; BGB, belowground biomass; TB, total biomass.

The correlations between species diversity and plant biomass in *D. purpurea* wetland communities are shown in Figure 4. The Shannon–Wiener index, Simpson’s diversity index, Pielou’s evenness index, and Patrick’s richness index all exhibited significant negative correlations with aboveground biomass (*p* < 0.01) and total biomass (*p* < 0.05), while no significant correlations were found with belowground biomass (*p* > 0.05). Additionally, aboveground, belowground, and total biomass were all significantly positively correlated with each other (*p* < 0.001).

### 2.4. Characteristics of Soil Physicochemical Properties Under Different Degradation Stages of D. purpurea Wetlands

Wetland degradation significantly influenced soil physicochemical properties (*p* < 0.05; Table 3). As degradation intensified, soil water content (WC), pH, available phosphorus (AP), total phosphorus (TP), and available potassium (AK) all showed significant decreasing trends, with non-degraded (ND) wetlands exhibiting significantly higher values than all degraded wetlands (*p* < 0.05). In contrast, dissolved organic nitrogen (DON) and ammonium nitrogen (NH_4_^+^-N) increased significantly with degradation, with HD wetlands showing the highest concentrations compared to other degradation stages (*p* < 0.05). Dissolved organic carbon (DOC) and nitrate nitrogen (NO_3_^−^-N) did not differ significantly among degradation gradients (*p* > 0.05), indicating that these factors are relatively insensitive to wetland degradation.

### 2.5. Relationships Between Plant Community Diversity, Biomass, and Soil Factors

**Figure 5:**
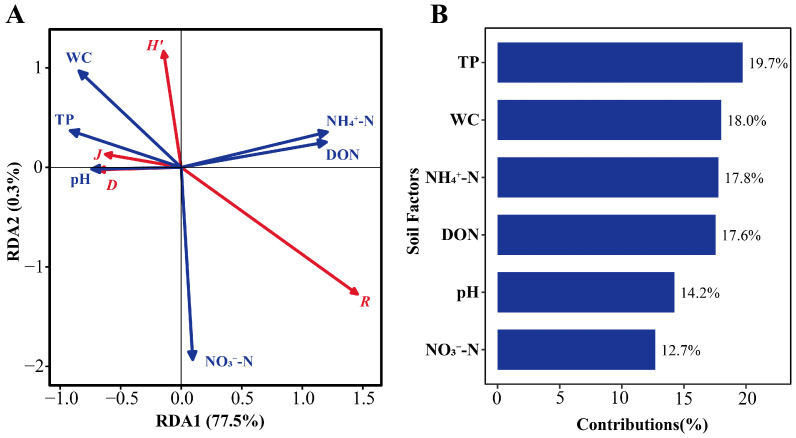
(**A**) Redundancy analysis (RDA) ordination of relationships between plant diversity and soil environmental factors; (**B**) contribution values of soil environmental factors to changes in plant diversity. *H′*, Shannon–Wiener index; *D*, Simpson index; *J*, Pielou index; *R*, Patrick index; TP, total phosphorus; NH_4_^+^-N, ammonium nitrogen; DON, dissolved organic nitrogen; WC, soil water content; pH, soil pH; NO_3_^−^-N, nitrate nitrogen.

The results of plant community diversity analysis and RDA of soil factors in *D. purpurea* wetlands are shown in Figure 5A. The first two RDA axes together explained 77.8% of the variation in plant community diversity. Soil pH, WC, TP, DON, and NH_4_^+^-N were identified as the primary factors driving changes in plant community diversity, accounting for 14.2%, 18.0%, 19.7%, 17.6%, and 17.8% of the variance, respectively (Figure 5B). All these factors significantly influenced plant community diversity (*p* < 0.001; Table 4). Specifically, the Shannon–Wiener index was negatively affected by nitrate nitrogen (NO_3_^−^-N). The Simpson and Pielou indices were positively influenced by soil pH, and TP, but negatively affected by NH_4_^+^-N and DON. Patrick’s richness index was primarily negatively influenced by soil WC (Figure 5A).

**Figure 6:**
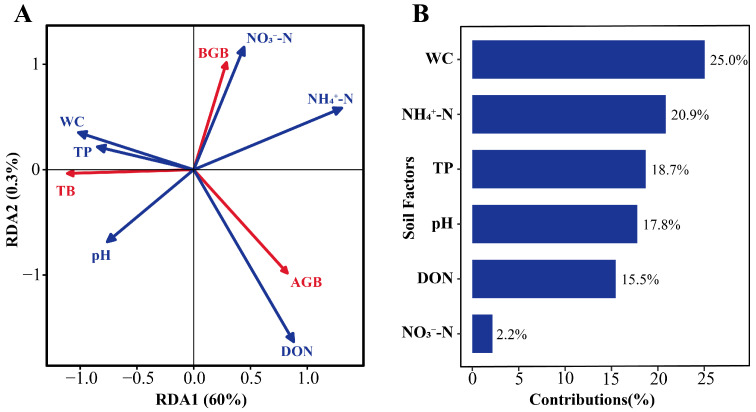
(**A**) Redundancy analysis (RDA) ordination of relationships between plant community biomass and soil environmental factors; (**B**) contribution values of soil environmental factors to changes in plant biomass. AGB, aboveground biomass; BGB, belowground biomass; TB, total biomass; WC, soil water content; NH_4_^+^-N, ammonia nitrogen; TP, total phosphorus; pH, soil pH; DON, dissolved organic nitrogen; NO_3_^−^-N, nitrate nitrogen.

The results of the RDA examining plant community biomass and soil factors in *D. purpurea* wetlands are shown in Figure 6A. The first two axes together explained 60.3% of the variation in plant community biomass. Soil WC, TP, and NH_4_^+^-N were identified as the primary factors influencing plant biomass, accounting for 25.0%, 18.7%, and 20.9% of the variance, respectively (Figure 6B). All these factors had highly significant effects on plant biomass (*p* < 0.001). Additionally, pH (*p* < 0.01) and DON (*p* < 0.05) also significantly affected biomass (Table 5). Specifically, aboveground biomass was positively influenced by DON but negatively affected by soil WC and TP. Belowground biomass was positively influenced by nitrate nitrogen (NO_3_^−^-N) and negatively affected by soil pH. Total biomass showed a negative response toNH_4_^+^-N (Figure 6A).

### 2.6. Effects of Core Soil Factors on Plant Community Characteristics

**Figure 7:**
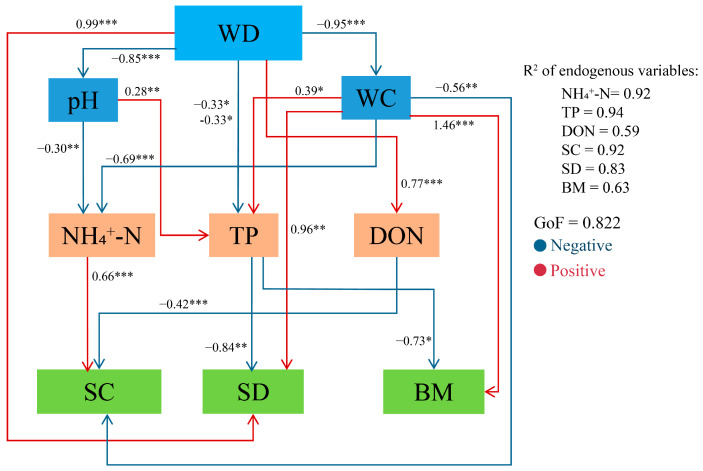
A structural equation model illustrating the effects of environmental factors on plant species diversity and biomass. Solid lines indicate significant paths, while dashed lines indicate non-significant paths. Blue and red lines represent negative and positive effects, respectively. The numbers on the arrows show standardized path coefficients. GOF, goodness-of-fit of the model. WD, wetland degradation; pH, soil pH; WC, soil water content; NH_4_^+^-N, ammonia nitrogen content; TP, total phosphorus content; DON, dissolved organic nitrogen content; SC, species composition; SD, diversity; BM, biomass. * *p* < 0.05; ** *p* < 0.01; *** *p* < 0.001.

To further investigate these relationships, the most representative core soil factors were selected, and partial least squares path modeling (PLS-PM) was employed to evaluate the effects of wetland degradation and key soil factors on plant diversity and biomass. The results are shown in Figure 7. The goodness-of-fit (GOF) value of the PLS-PM model was 0.822, indicating a robust model fit. Specifically, wetland degradation had a direct positive effect on plant diversity (path coefficient = 0.99, *p* < 0.001). Soil WC exerted a direct positive effect on plant biomass (path coefficient = 1.46, (*p* < 0.001) and a direct negative effect on plant community species composition (path coefficient = −0.56, *p* < 0.01). Ammonium nitrogen (NH_4_^+^-N) showed a strong direct positive effect on plant community species composition (path coefficient = 0.66, *p* < 0.001), while TP negatively influenced plant community diversity (path coefficient = −0.84, *p* < 0.01). DON directly negatively affected plant community species composition (path coefficient = −0.42, *p* < 0.001). Intensified wetland degradation significantly increased DON content, which indirectly reduced plant community species composition. Decreases in soil pH and WC elevated NH4^+^-N levels, indirectly enhancing plant community species composition while reducing TP content, thereby increasing plant community diversity. Furthermore, the coefficients of determination (R^2^) for soil NH_4_^+^-N, TP content, plant community species composition (SC), diversity (SD), and biomass (BM) were 0.92, 0.94, 0.92, 0.83, and 0.63, respectively. All R^2^ values exceeded 0.6, demonstrating the strong explanatory power of the model.

**Figure 8:**
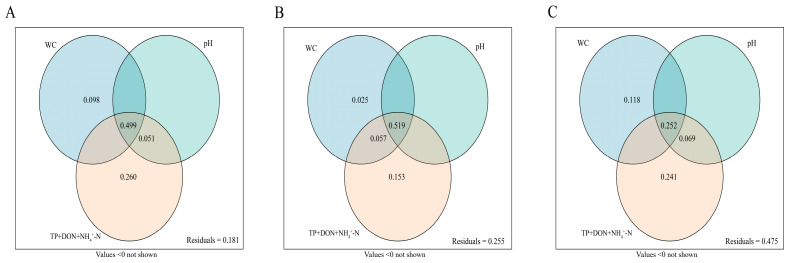
The results of variation partitioning analyses illustrating the relative contribution of WC; pH and TP + DON + NH_4_^+^-N in (**A**) Species Composition, (**B**) Species Diversity and (**C**) Biomass. In each panel, the non-overlapping areas of the circles denote the pure effects of each variable (WC, pH, and TP + DON + NH_4_^+^-N, respectively), while the overlapping areas represent the joint effects of multiple variables. “Residuals” refers to the proportion of variation in the corresponding ecological index (Species Composition, Species Diversity, or Biomass) that cannot be explained by these three groups of variables. Key finding: species composition is dominated by the joint effect of WC, pH and nutrients (49.9%), species diversity is primarily driven by the joint effect of WC, pH, and nutrients (51.9%), and biomass relies on both the independent effect of nutrients (24.1%) and the joint effect of WC, pH and nutrients (25.2%).Abbreviations: WC, Water Content; TP, Total Phosphorus; DON, Dissolved Organic Nitrogen; NH_4_^+^-N, Ammonium Nitrogen.

To disentangle the regulatory contributions of soil WC, pH, and composite nutrient factors (TP, DON, and NH_4_^+^-N) to plant community traits in *Deyeuxia purpurea* wetlands, variance partitioning analysis (VPA) was performed, and the results (Figure 8) demonstrated the following: these three factor groups explained 81.9% of the total variation in species composition (Figure 8A), where the joint effect of all three factors (49.9%) served as the largest contributor, followed by the pure effects of nutrient factors (26.0%) and WC (9.9%), with a residual variation of only 18.1%; for species diversity (Figure 8B), the total explained variation reached 74.5%, dominated by the WC-pH joint effect (51.9%) and supplemented by the pure effects of nutrients (15.3%) and WC (11.8%), with a residual variation of 25.5%; for biomass (Figure 8C), the total explained variation was only 52.5%, with WC’s pure effect (24.1%) acting as the core driver, followed by the WC-nutrient joint effect (25.2%), and a residual variation of 47.5%. Overall, species composition was dominated by multi-factor synergies, diversity centered on WC-pH interactions, and biomass was independently regulated by WC, with distinct dominant regulatory pathways across different traits.

## 3. Discussion

### 3.1. Effects of D. purpurea Wetland Degradation on Plant Community Characteristics

The dynamic characteristics of plant communities are essential for assessing the health of local ecosystems, and examining plant ecological traits is a key step in evaluating the restoration progress of degraded wetlands [28]. Wetland degradation induces substantial changes in plant community composition and diversity [29]. Our results indicate that degradation was significantly associated with the succession of plant communities in *D. purpurea* wetlands on the Sanjiang Plain (Table 1, Figure 1). However, these patterns are inferred from observational data along a spatial gradient, and we caution that unmeasured factors, such as subtle hydrological variations or historical disturbances, could contribute to differences in species composition. As degradation intensified, the dominance of wetland pioneer species such as *D. purpurea* gradually declined. Companion species shifted from wetland-adapted plants, such as Phragmites australis, to intermediate species like *Lysimachia thyrsiflora* (LD) and *Stellaria radians* (MD), and finally to semi-xerophytic species such as *Geranium platyanthum* and *Onoclea sensibilis* var. *interrupta* (HD). These findings align with Zhang et al. [30], who observed a similar succession pattern in which plant communities shifted from hydrophilous to mesophilous and then to xerophilous species during the degradation of *Sparganium ruber* in the Momoge Wetland, suggesting a common trajectory in freshwater marsh wetland degradation, albeit potentially modulated by site-specific hydrology. NMDS analysis in this study is employed to illustrate succession pattern trajectories and reveals that community structural heterogeneity peaks in the severely degraded stage. This pattern is closely correlated with increased microhabitat fragmentation, altered hydrological conditions, and changes in soil physicochemical properties, with plant community composition largely associated with habitat differentiation [31].

Plant species diversity is critical for ecosystem function and serves as an indicator of ecosystem stability [32]. Our study found that the Shannon–Wiener index, Simpson’s diversity index, Pielou’s evenness index, and Patrick’s richness index all increased significantly with wetland degradation (Figure 2). While this may appear to contradict general wetland theory where degradation often reduces diversity [15,33], it is consistent with patterns in transitional degradation stages, where invasion by generalist mesophytic and xerophytic species (e.g., *Geranium platyanthum*) increases heterogeneity and richness [13,15]. These results are consistent with previous studies by Wu et al. [15], indicating that degradation drives a shift in plant communities from aquatic/hydrophytic species (e.g., *D. purpurea*) toward mesophytic and xerophytic species. The Sorenson similarity index decreased from 66.67% in ND-LD wetlands to 14.63% in ND-HD wetlands (Table 2), confirming that this pattern reflects genuine species replacement rather than changes in the abundance of existing species. Overall, wetland plant diversity increased with degradation to a certain extent, likely due to increased habitat heterogeneity and invasion by generalists, but this represents functional decline rather than improvement. This pattern aligns with Ye et al. [34] and with An et al. [33], who reported a decline in diversity as degraded wetlands undergo restoration, with diversity gradually approaching that of native wetlands. This reflects the reverse-succession characteristic of the degradation–restoration continuum: degradation shifts the community from a native wetland state toward xerophytic weeds, while restoration moves from xerophytic weeds to wetland pioneer species and ultimately toward the natural wetland background species, with community characteristics and functions progressively returning to their original state [35]. This bidirectional succession explains the opposite trends in species diversity observed between degraded and restored wetlands, supporting the rationale of this study.

### 3.2. Relationship Between Species Diversity and Plant Biomass

Biomass is not only a central measure of ecosystem structure and function but also a key indicator of productivity and carbon cycling capacity. Plant biomass is closely linked to species diversity, although the nature of this relationship varies depending on environmental gradients and species traits, and has not been consistently defined in the literature. In this study, plant diversity indices in *D. purpurea* wetland communities were significantly negatively correlated with both aboveground and total biomass (Figure 4). To explore whether this reflects dominance effects rather than degradation-induced loss, we examined species-level biomass contributions (Supplementary Table S1). In ND wetlands, *D. purpurea* accounted for 93.68% of total biomass, suppressing diversity through competitive dominance. As degradation intensified, its contribution declined to 18.08% in HD sites, enabling invasion by mesophytic species and increasing diversity, but at the cost of reduced overall biomass. These results partially align with Huang et al. [36], who found that in floodplain wetlands of Dongting Lake, vegetation biomass increased with rising Patrick’s richness and Shannon–Wiener indices in the *Persicaria hydropiper* community, but decreased with increasing diversity in *Carex brevicuspis* and *Miscanthus sacchariflorus* communities. ND wetlands in the Sanjiang Plain exhibited the highest biomass but relatively low species richness. This pattern is primarily associated with soil physicochemical properties and moisture conditions. Early-stage degradation, characterized by water loss, nutrient depletion, and soil compaction, limits vegetation growth. Dominant species like *D. purpurea* show reduced height, coverage, and density, causing sharp declines in overall vegetation biomass [19]. Although plant diversity in ND wetlands did not differ significantly from that in LD wetlands, it was markedly lower than in MD and HD wetlands. This pattern aligns with the vegetation-soil positive feedback mechanism: vigorous growth of dominant species produces substantial aboveground and belowground biomass, which returns organic matter to the soil through litterfall. This enhances SOC and nutrient content, maintaining favorable soil structure and moisture that further support plant growth [37]. Overall, plant diversity and biomass were negatively correlated in this study. During wetland degradation, species turnover significantly affects community composition, diversity, and productivity. As degradation intensifies, plant communities shift from hydrophytic to drought-tolerant or mesophytic species, resulting in reduced vegetation cover, lower biomass, and decreased ecosystem productivity [38].

### 3.3. Relationship Between Soil Physicochemical Properties and Plant Community Characteristics

Wetland degradation primarily manifests as soil water loss, which in turn affects nutrient availability and overall soil physicochemical properties, ultimately altering plant community characteristics [39]. RDA and Mantel tests revealed that the soil factors driving plant community diversity differed from those influencing plant biomass in D. purpurea wetlands. Specifically, soil pH, WC, TP, DON, and NH_4_^+^-N were key drivers of community diversity, whereas pH, WC, TP, and NH_4_^+^-N primarily affected plant biomass. Across both diversity and biomass, soil WC and pH were identified as the most critical factors. Soil WC—measured independently via gravimetric methods—exhibits strong correlations with plant growth and distribution, which is consistent with known ecological mechanisms: reduced water availability can constrain plant water acquisition and nutrient mobility, a pattern that aligns with the observed changes in community composition and diversity during early degradation [40]. This avoids circularity, as WC was not inferred from vegetation patterns but quantified separately. Furthermore, soil pH indirectly regulates plant growth and community traits by influencing nutrient availability, microbial activity, and root absorption, with its effects becoming more pronounced once water is no longer the primary limiting factor [41,42]. In this study, Simpson’s diversity index and Pielou’s evenness index of plant communities were positively correlated with TP. Meng et al. [43] suggested that soils with higher TP content provide sufficient phosphorus resources to support more plant species, thereby reducing competitive exclusion and decreasing the dominance of particular species. In contrast, AP and AK had limited effects on vegetation diversity, showing insignificant correlations. This indicates that AP and AK are not primary determinants of wetland plant diversity. Similarly, Zhang et al. [44] reported that AK and AP were significantly positively correlated with plant biomass but had minimal influence on community diversity. Notably, soil indicators such as TP and TK exhibited stronger correlations with plant diversity than available nutrients, which may reflect either the non-limiting nature of nutrients in wetland ecosystems or threshold responses of plants to nutrient availability [45]. Nitrogen sources, NH_4_^+^-N and DON, can meet plant nutritional requirements and promote species coexistence by regulating nitrogen cycling [46,47]. In this study, both NH_4_^+^-N and DON significantly influenced plant community diversity and biomass, showing positive correlations with belowground and aboveground biomass, respectively, highlighting the importance of nitrogen availability for sustaining wetland plant communities. In contrast, DOC had no significant effect on community traits, likely because DOC is highly labile and preferentially consumed by microorganisms, rendering its regulatory role in soil transient and limited [48].

This study employed a combination of partial least squares path modeling (PLS-PM) and variance partitioning analysis (VPA) to examine the causal relationships among wetland degradation, key soil factors, and plant community species composition, diversity, and biomass (Figure 7 and Figure 8). The results indicate that wetland degradation directly or indirectly influences both critical soil physicochemical factors and plant community characteristics; consistent with the findings of Wan et al. [13], our findings suggest that wetland degradation does not produce uniform effects on soil and plant communities. These differences may arise from variations in soil types across freshwater marshes, riverine wetlands, and inland salt marshes, or from differences in ecosystem responses to degradation. Specifically, soil WC exhibited a direct positive effect on plant biomass but a direct negative effect on species composition. Higher soil moisture favors hydrophytic plant growth, increasing overall biomass, as elevated moisture promotes root expansion and belowground biomass accumulation. Simultaneously, increased WC allows moisture-tolerant dominant species to outcompete others, leading to a more homogeneous species composition. Plant community species composition was also directly positively influenced by ammonium nitrogen (NH_4_^+^-N) and negatively influenced by DON. Elevated NH_4_^+^-N enhances the growth and competitiveness of nitrogen-demanding species, allowing them to dominate the community and accumulate biomass [49]. Conversely, in environments with high DON, nutrient-demanding species are less competitive, promoting greater community diversity [50]. In summary, the relationships between wetland plant communities and key soil physicochemical factors are dynamic and influenced by degradation. The reciprocal interactions observed highlight that plant–soil feedbacks play a central role in wetland degradation. While clarifying the plant–soil association characteristics during the degradation of *D. purpurea* wetlands in the Sanjiang Plain, this study has certain limitations. Conducted only based on a single degradation gradient, it focused on the apparent correlations between plants and soils without incorporating microbial communities, the lack of consideration of their role limits the comprehensive interpretation of the intrinsic mechanisms underlying plant–soil interactions. Differences in species composition are more likely the combined result of degradation processes, unquantified hydrological factors, and historical disturbance events, whose potential impacts require further clarification. Additionally, the analysis of plant communities was confined to apparent indicators, failing to address intrinsic functional attributes and thus insufficiently elaborating on community assembly mechanisms.

Building on these limitations, future research can be targeted and advanced: introduce the perspective of plant functional traits, quantify key traits such as drought tolerance and nutrient use efficiency, analyze the regulatory mechanisms of species functional strategies on the biomass–diversity relationship, and reveal the intrinsic logic of community assembly under degradation stress. Simultaneously, expand the research dimension to construct plant–soil-microbial analytical framework, combine multi-gradient experiments with long-term monitoring to distinguish the independent and interactive effects of various factors, clarify the mediating role of microorganisms, and verify the generalizability of conclusions, thereby providing precise theoretical support for the ecological restoration of degraded wetlands.

## 4. Materials and Methods

### 4.1. Study Area

**Figure 9:**
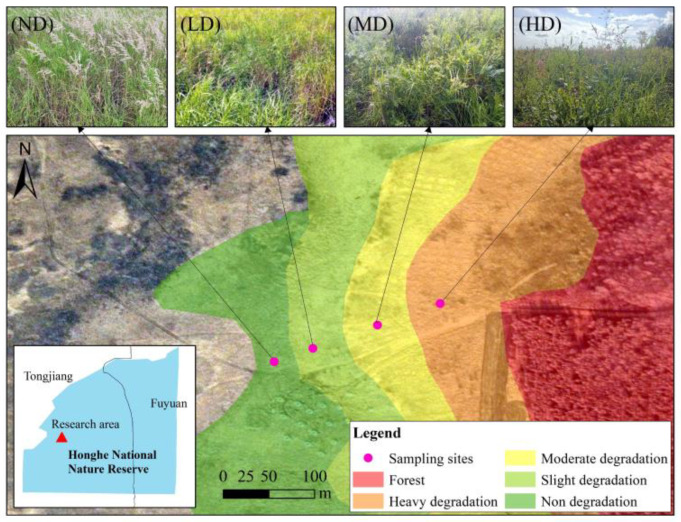
Location of study area and distribution of sampling sites. ND, non-degraded; LD, slightly degraded; MD, moderately degraded; HD, heavily degraded.

The study was conducted at the Sanjiang Plain Wetland Ecological Research Station of the Institute of Natural Resources and Ecology, Heilongjiang Academy of Sciences, located within the Honghe National Nature Reserve. The reserve is located in the northeastern Sanjiang Plain, Heilongjiang Province, China (47°42′–47°52′ N, 133°34′38″–133°46′29″ E). The region has a temperate monsoon climate, with a mean annual temperature of 1.9 °C and elevations ranging from 50 to 60 m. The average annual precipitation is approximately 585 mm, of which 50–70% occurs from July to September (Figure 9). The dominant soil types are Albic soils and marsh soils, and the vegetation is mainly composed of meadow and marsh communities [27].

### 4.2. Field Sampling

*D. purpurea* in the Sanjiang Plain begins to grow in May, with aboveground biomass reaching its peak in late July. We conducted a vegetation survey in July 2024 using the methods of Wan et al. [13] and Yang et al. [51]. Transects were established extending outward from the wetland core area based on water level gradients and vegetation conditions. Degraded wetlands were preliminarily classified as slightly degraded (LD), moderately degraded (MD), and heavily degraded (HD) according to *Deyeuxia purpurea* cover, while naturally non-degraded wetlands (ND) were selected as controls (Table 6). All degraded and non-degraded wetlands experienced similar climatic conditions. The categorization of these stages was based primarily on the characteristics of vegetation composition, with each stage typically separated by an approximate distance of 50 m, reflecting a gradient in vegetation transition and wetland health. Through data investigation, we have confirmed that all the points on the sampled line were swamp wetlands 20 years ago, which ensures that the spatial sampling can represent vegetation degradation. For each degradation level, nine 1 m × 1 m sampling plots were randomly selected to assess species richness and measure plant height, cover, and density of each species. To ensure plot independence by eliminating interference from *D. purpurea* clonal reproduction (rhizome expansion) and lateral soil nutrient migration, a minimum 5 m buffer zone was established between any two plots, with no sampling quadrats allocated therein. Within each 1 m × 1 m vegetation plot, one 50 cm × 50 cm subplot was randomly chosen for aboveground biomass harvesting. Belowground biomass was sampled using a root sampler (10 cm × 10 cm cross-section, 30 cm depth).

Soil samples were collected from the 0–30 cm layer using a soil sampler. After collection, the top 0–10 cm humus layer was removed, and the remaining 10–30 cm soil (20 cm in total) was retained for subsequent analysis. This treatment was conducted for three key reasons: First, the top 0–10 cm layer is dominated by fresh and semi-decomposed plant litter, with highly variable physicochemical properties that could mask the long-term effects of wetland degradation on soil quality. Second, *D. purpurea*, the dominant species in the study area, develops a fibrous root system primarily distributed in the 10–30 cm soil layer, where root–soil interactions (e.g., nutrient absorption and rhizosphere processes) are most intensive; thus, this layer better reflects the soil environmental conditions directly affecting plant growth and community dynamics. Third, excluding the surface humus layer avoids interference from non-soil components and reduces experimental variability and comparability of soil physicochemical property data across different degradation stages. All soil samples were sealed, stored at 4 °C, and transported to the laboratory together with the plant samples for subsequent analyses.

### 4.3. Analysis of Soil and Plant Samples

Plant samples were transported to the laboratory, washed, and separated by component. They were then dried at 65 °C to constant weight and weighed to determine biomass. Fresh soil samples were carefully cleared of plant roots and stones. One portion was oven-dried to determine soil moisture content, while the remaining portion was air-dried, ground, and passed through a 100-mesh sieve to remove residual roots and stones for subsequent physicochemical analyses. Soil pH was measured using the potentiometric method (METTLER TOLEDO, Greifensee, Switzerland). Total phosphorus was determined using the NaOH fusion-molybdenum antimony colorimetric method, and available phosphorus was measured by extraction with 0.05 mol/L HCl–0.025 mol/L H_2_SO_4_ followed by molybdenum–antimony colorimetry. Total potassium was analyzed using the NaOH fusion–flame photometric method, whereas available potassium was determined by NH_4_OAc extraction followed by flame photometry. Soluble organic carbon and soluble organic nitrogen were extracted with 0.5% calcium chloride and quantified using a total organic carbon-nitrogen analyzer [52]. Ammonium nitrogen and nitrate nitrogen were extracted by shaking soil samples with 2 mol/L KCl solution for 1 h and subsequently measured using an AA3 continuous-flow chemical analyzer [27].

### 4.4. Calculation of Community Diversity Indices

To characterize changes in plant communities during wetland degradation relative to non-degraded conditions, the importance value (IV) was used to represent the relative contribution of each plant species within the community. Species diversity was evaluated using the Shannon–Wiener diversity index (H′), Simpson’s dominance index (D), Patrick’s richness index (R), and Pielou’s evenness index (J). Sørensen’s similarity index (I) was applied to assess the similarity between degraded wetland communities and non-degraded wetland communities.

(1) Importance value (IV):
  (1) $$IV=\frac{RD+RH+RC}{3}\times 100$$

In Equation (1), RD represents relative density, RH represents relative height, and RC represents relative coverage.

(2) Shannon–Wiener diversity index (*H′*):
  (2) $$H^{\prime }=-\sum_{i=1}^{s}P_{i}lnP_{i},$$

In Equation (2), P_i_ = Ni/N, where Ni is number of individuals of species i, N is the total number of individuals of all species in the quadrat, and S is the total number of species in the quadrat.

(3) Simpson’s dominance index (*D*):
  (3) $$D=1-\sum_{i=1}^{S}{P_{i}}^{2},$$

In Equation (3), P_i_ = Ni/N, where Ni is number of individuals of species i, N is the total number of individuals of all species in the quadrat, and S is the total number of species in the quadrat.

(4) Patrick richness index (*R*)
  (4) R=S,

In Equation (4), S is the total number of species in the 1 m^2^ quadrat.

(5) Pielou’s evenness index (*J*):

In Equation (5), H′ is the Shannon–Wiener diversity index, and S is Patrick’s richness index.

(5) $$J=\frac{H\prime }{{ln}^{s}},$$

(6) Sorenson’s similarity index (I):
  (6) $$I=\frac{2c}{a+b}\times 100\%$$

In Equation (6), a is the number of species in the degraded wetland, b is the number of species in the non-degraded wetland, and c is the number of species shared by both the degraded and non-degraded wetlands.

### 4.5. Data Processing

Data analysis and graphing were performed using Origin 2018 (OriginLab Corporation, Northampton, MA, USA), IBM SPSS Statistics 25.0 (IBM Corp., Armonk, NY, USA), and R 4.5.1 (R Core Team, Vienna, Austria). In SPSS 25.0, species diversity indices, biomass, and soil physicochemical properties were first assessed for normality and homogeneity of variance, with all variables conforming to the normal distribution and homogeneity assumptions; these parameters were then analyzed using one-way ANOVA and Tukey’s test, with the results expressed as means ± standard errors. To address potential spatial autocorrelation, Moran’s I was calculated on ANOVA residuals using the ‘spdep’ package (version 1.3.3) in R, confirming no significant autocorrelation (*p* > 0.05). Additionally, linear mixed-effects models (lme4 package) were applied with transect as a random effect to account for any hierarchical structure, yielding consistent results with ANOVA. To account for multiple testing, false discovery rate (FDR) correction was applied using the Benjamini–Hochberg method across ANOVA post hoc tests, Mantel tests, and RDA permutations.

Non-metric multidimensional scaling (NMDS) was conducted using the ‘vegan’ package (version 2.6.6.1) in R 4.5.1, indicator species analysis was performed with the ‘indicspecies’ package (version 1.7.14), and correlation heatmaps were generated using the ‘ggally’ package (version 2.2.1). Redundancy analysis (RDA) was employed to evaluate relationships between plant community diversity/biomass and soil factors. To avoid overfitting, forward selection (‘ordistep’ in vegan) was applied with FDR-corrected *p*-values (*p* < 0.05), reducing variables while checking VIF (<5). To quantify the independent and joint explanatory contributions of different soil factor groups to variations in plant community traits, variance partitioning analysis (VPA) was further implemented using the ‘varpart’ function in the vegan package. Mantel tests were used to assess the explanatory power of variables, and the most representative core soil factors (i.e., those significantly influencing both plant community diversity and biomass while offering the highest explanatory capacity) were selected following Fan et al. [53]. The plspm package (version 0.2.1) in R was used to construct partial least squares path models, quantifying the direct and indirect effects of wetland degradation and key soil factors on plant diversity and biomass to elucidate underlying driving mechanisms.

## 5. Conclusions

Wetland degradation is a key driver of succession in plant communities, with clear differences observed across degradation levels. Degradation shifts plant communities from dominance by a single species to the coexistence of multiple species, accompanied by a habitat transition from hydrophytic to mesophytic and xerophytic conditions, and the invasion of mesophytic and xerophytic weeds, ultimately impairing wetland ecological functions. Aboveground, belowground, and total plant biomass decline progressively with degradation, while plant diversity shows a significant negative correlation with biomass. Intensified degradation directly increases plant community diversity and indirectly alters species composition through key soil factors, including soil pH, WC, NH_4_^+^-N, and DON. Soil WC emerges as a critical driver, positively promoting plant biomass accumulation while contributing to species homogenization. Soil pH indirectly influences community composition, diversity, and biomass by regulating NH_4_^+^-N and TP. While PLS-PM quantifies relationships among degradation, soil factors, and plant characteristics, causality inferences are limited by the cross-sectional design. Longitudinal studies are recommended to confirm these pathways. This study clarifies the vegetation responses and environmental drivers associated with *D. purpurea* wetland degradation. However, due to the complexity of these mechanisms, further research is needed to explore additional influencing factors in degraded wetlands. Given that soil WC and NH_4_^+^-N were identified as key regulators of plant community characteristics, interventions in the Sanjiang Plain should implement differentiated management strategies: prioritize maintaining soil moisture (target 70–80%) and supplementing organic matter for slightly degraded wetlands; intensify regular water supplementation (when WC < 60%) and apply ammonium-based fertilizers for moderately degraded wetlands; and intensify water restoration, supplement nitrogen, and add phosphorus-rich organic amendments for heavily degraded wetlands to support *D. purpurea* populations and suppress the invasion of mesophytic/xerophytic weeds.

## Figures and Tables

**Table 1:** Dominant plant importance values (IVs) and indicator species in *D. purpurea* communities of the Sanjiang Plain wetlands along a degradation gradient.

Species	Abbrev.	ND	LD	MD	HD
*Deyeuxia purpurea*	Depu	660.46	551.62	453.41	182.88
*Viola mandshurica*	Vima	—	—	3.78 (0.39 NS)	—
*Galium dahuricum* var. *lasiocarpum*	Gada	—	—	70.95	97.65
*Anemone dichotoma*	Andi	—	113.50	102.07	11.23
*Lathyrus quinquenervius*	Laqu	15.83	10.67	20.90	2.47
*Veratrum dahuricum*	Veda	—	—	—	27.58 (0.60 **)
*Sanguisorba officinalis*	Saof	—	—	4.10	9.26 (0.32 NS)
*Hypericum japonicum*	Hyja	—	—	2.71 (0.29 NS)	—
*Stellaria radians*	Stra	—	—	42.63 (0.78 ***)	—
*Anemone udensis*	Anud	—	—	—	2.55 (0.29 NS)
*Galium kamtschaticum*	Gaka	—	—	—	16.96 (0.66 ***)
*Sanguisorba tenuifolia* var. *alba*	Sate	—	—	—	4.26 (0.29 NS)
*Vicia bungei*	Vibu	—	8.93	—	6.25
*Deyeuxia neglecta*	Dene	—	5.28	—	62.94 (0.73 ***)
*Persicaria thunbergii*	Peth	21.09	19.20	—	—
*Poa annua*	Poan	—	—	—	33.76 (0.42 NS)
*Euphorbia lucorum*	Eulu	—	—	—	1.63 (0.29 NS)
*Equisetum sylvaticum*	Eqsy	—	—	—	4.91 (0.41 NS)
*Carex miyabei* var. *maopengensis*	Cami	65.02 (0.66 ***)	29.60	14.57	—
*Stachys baicalensis*	Stba	—	3.64 (0.29 NS)	—	—
*Geranium platyanthum*	Gepl	—	—	—	67.98 (0.93 ***)
*Thelypteris palustris*	Thpa	—	—	—	59.39 (0.87 ***)
*Epilobium palustre*	Eppa	—	3.59	1.78	2.45
*Iris sanguinea*	Irsa	—	—	—	2.84 (0.29 NS)
*Carex pseudo-conica*	Caps	19.72 (0.59 **)	—	—	—
*Carex appendiculata*	Caap	48.73	53.66	94.99	78.40
*Cirsium pendulum*	Cipe	—	—	7.73 (0.29 NS)	—
*Glyceria spiculosa*	Glsp	52.79	56.59	—	—
*Onoclea sensibilis* var. *interrupta*	Onse	—	—	—	110.56 (0.88 ***)
*Lysimachia thyrsiflora*	Lyth	—	23.39 (0.76 ***)	—	—
*Viola lactiflora*	Vila	—	—	—	4.70 (0.42 NS)
*Persicaria perfoliata*	Pepe	—	—	—	4.09 (0.29 NS)
*Thalictrum simplex*	Thsi	—	—	20.96	20.36
*Scutellaria dependens*	Scde	—	—	3.70 (0.29 NS)	—
*Galium trifidum*	Gatr	14.52 (0.60 ***)	3.54	—	—
*Vicia tenuifolia*	Vite	—	—	—	1.44 (0.29 NS)
*Filipendula × intermedia*	Fiin	—	—	—	41.12 (0.73 **)
*Thalictrum aquilegiifolium*	Thaq	—	—	—	2.11 (0.29 NS)
*Phragmites australis*	Phau	—	—	—	13.58 (0.40 NS)
*Saussurea amara*	Saam	—	—	—	4.47 (0.41 NS)
*Moehringia lateriflora*	Mola	—	11.69	16.21	5.79
*Pseudolysimachion longifolium*	Pslo	—	—	—	4.68 (0.39 NS)
*Hypericum longistylum*	Hylo	1.83	5.10	4.63	—
*Saussurea japonica*	Saja	—	—	2.51	9.70 (0.35 NS)
*Nelumbo lutea*	Nelu	—	—	32.39 (0.67 **)	2.01

ND, non-degraded; LD, slightly degraded; MD, moderately degraded; HD, heavily degraded. Indicator values are shown in parentheses. *** *p* < 0.001; ** *p* < 0.01; NS, no significance; —, indicates the absence of the species.

**Table 2:** Similarity analysis of degraded wetland plant communities.

Degradation Stage	ND	LD	MD	HD
ND	—	—	—	—
LD	66.67%	—	—	—
MD	35.71%	47.06%	—	—
HD	14.63%	34.04%	43.14%	—

ND, non-degradation; LD, slight degradation; MD, moderate degradation; HD, heavy degradation; —, indicates the absence of the species.

**Table 3:** Soil physical and chemical properties in *D. purpurea* wetlands at different degradation stages.

Factor	ND	LD	MD	HD
pH	4.76 ± 0.06 a	4.47 ± 0.03 b	4.44 ± 0.02 b	4.18 ± 0.00 c
AP (mg·kg^−1^)	59.76 ± 2.33 a	49.82 ± 1.35 b	41.71 ± 1.16 c	54.17 ± 1.17 b
TP (g·kg^−1^)	18.36 ± 0.12 a	15.22 ± 0.14 b	13.85 ± 0.23 c	10.38 ± 0.22 d
TK (g·kg^−1^)	20.12 ± 0.18 d	23.07 ± 0.45 b	26.82 ± 0.15 a	21.71 ± 0.10 c
AK (mg·kg^−1^)	216.81 ± 2.23 a	90.09 ± 0.81 d	104.38 ± 1.90 c	152.52 ± 0.88 b
DOC (mg·kg^−1^)	1240.25 ± 112.95 a	1297.57 ± 155.43 a	1440.30 ± 60.06 a	1417.02 ± 41.58 a
DON (mg·kg^−1^)	23.13 ± 1.23 c	22.84 ± 0.75 c	28.22 ± 0.74 b	32.83 ± 0.60 a
NO_3_^−^-N (mg·kg^−1^)	1.01 ± 0.03 a	1.27 ± 0.08 a	1.16 ± 0.65 a	1.09 ± 0.04 a
NH_4_^+^-N (mg·kg^−1^)	7.16 ± 0.09 d	19.88 ± 0.13 b	19.05 ± 0.19 c	29.88 ± 0.33 a
WC (%)	118.54 ± 0.06 a	74.57 ± 0.05 b	55.69 ± 0.04 c	37.65 ± 0.03 d

ND, non-degradation; LD, slight degradation; MD, moderate degradation; HD, heavy degradation; pH, soil pH; AP, available phosphorus; TP, total phosphorus; TK, total potassium; AK, available potassium; DOC, dissolved organic carbon; DON, dissolved organic nitrogen; NO_3_^−^-N, nitrate nitrogen; NH_4_^+^-N, ammonia nitrogen; WC, soil water content. Data are presented as mean ± standard error (SE). Different letters indicate significant differences between treatments (*p* < 0.05).

**Table 4:** Mantel test for correlations between plant community diversity and environmental factors.

Factor	R^2^	FDR-Adjusted *p*
pH	0.4796	0.0012 **
WC	0.5961	0.0012 **
TP	0.6831	0.0012 **
DON	0.6062	0.0012 **
NH_4_^+^-N	0.6154	0.0012 **
NO_3_^−^-N	0.0055	0.677

pH, soil pH; TP, total phosphorus; DON, dissolved organic nitrogen; NO_3_^−^-N, nitrate nitrogen; NH_4_^+^-N, ammonia nitrogen; WC, soil water content. Significance levels: ** *p* < 0.01.

**Table 5:** Mantel test for correlations between plant community biomass and environmental factors.

Factor	R^2^	FDR-Adjusted *p*
pH	0.3312	0.003 **
WC	0.5291	0.002 **
TP	0.3939	0.002 **
NH_4_^+^-N	0.4389	0.002 **
DON	0.1680	0.012 *
NO_3_^−^-N	0.0216	0.426

pH, soil pH; TP, total phosphorus; DON, dissolved organic nitrogen; NO_3_^−^-N, nitrate nitrogen; NH_4_^+^-N, ammonia nitrogen; WC, soil water content. Significance levels: * *p* < 0.05, ** *p* < 0.01.

**Table 6:** Basic information of sampling sites.

Degradation Stage	*D. purpurea* Coverage	Basic Information
Non-degradation (ND)	>90%	Seasonal waterlogging occurs, with almost no dry patches
Slight degradation (LD)	60–80%	Seasonal waterlogging occurs, with a small number of scattered dry patches
Moderate degradation (MD)	30–50%	The duration and extent of waterlogging decrease, while the area of dry patches increases
Heavy degradation (HD)	10–20%	Almost no waterlogging, with extensive and continuous dry patches

## Data Availability

The original contributions presented in this study are included in the article. Further inquiries can be directed to the corresponding authors.

## Supplementary Materials

The following supporting information can be downloaded at: https://www.mdpi.com/article/10.3390/plants15060918/s1, Table S1: Biomass proportion of *D. purpurea* under different degradation stages. ND, non-degradation; LD, slight degradation; MD, moderate degradation; HD, heavy degradation; —, indicates the absence of the species. Data are presented as mean ± standard error (SE). Different letters indicate significant differences between treatments (*p* < 0.05).

## Abbreviations

The following abbreviations are used in this manuscript:

ND	Non-degraded
LD	Slightly degraded
MD	Moderately degraded
HD	Heavily degraded
*D. purpurea*	*Deyeuxia purpurea*
NMDS	Non-metric multidimensional scaling
RDA	Redundancy analysis
IV	Importance values
*H′*	Shannon–Wiener diversity index
*D*	Simpson dominance index
*R*	Patrick richness index
*J*	Pielou’s evenness index
I	Sorenson’s similarity index
AGB	Aboveground biomass
BGB	Belowground biomass
TB	Total biomass
pH	Soil pH
WC	Soil water content
TP	Total phosphorus
TK	Total potassium
AP	Available phosphorus
AK	Available potassium
NH_4_^+^-N	Ammonia nitrogen
NO_3_^−^-N	Nitrate nitrogen
DON	Dissolved organic nitrogen
DOC	Dissolved organic carbon
PLS-PM	Partial least squares path modeling
GOF	Goodness-of-fit
SC	Species composition
SD	Diversity
BM	Biomass
