# Supplementary material for: The Effects of Deyeuxia purpurea Wetland Degradation on Plant Communities and Key Soil Factors in the Sanjiang Plain
Source: Plants (Basel). 2026 Mar 16;15(6):918. doi: 10.3390/plants15060918 (PMC13029699; doi:10.3390/plants15060918)
Supplement: Supplementary file 1 [file plants-15-00918-s001.zip › plants-4087159-supplementary.pdf]

Table S1. Biomass proportion of *D. purpurea* under different degradation stages.

Biomass proportion (%)	ND	LD	MD	HD
D. purpurea	93.68 ± 0.90a	72.69 ± 1.35b	50.79 ± 1.47c	18.08 ± 0.93d

ND, non-degradation; LD, slight degradation; MD, moderate degradation; HD, heavy degradation; —, indicates the absence of the species. Data are presented as mean ± standard error (SE). Different letters indicate significant differences between treatments ($p < 0.05$).
